# Orientation Sensitivity at Different Stages of Object Processing: Evidence from Repetition Priming and Naming

**DOI:** 10.1371/journal.pone.0002256

**Published:** 2008-05-28

**Authors:** Irina M. Harris, Paul E. Dux, Claire T. Benito, E. Charles Leek

**Affiliations:** 1 School of Psychology, University of Sydney, Sydney, Australia; 2 Department of Psychology, Vanderbilt University, Nashville, Tennessee, United States of America; 3 School of Psychology, University of Wales, Bangor, United Kingdom; Ecole Polytechnique Federale de Lausanne, Switzerland

## Abstract

**Background:**

An ongoing debate in the object recognition literature centers on whether the shape representations used in recognition are coded in an orientation-dependent or orientation-invariant manner. In this study, we asked whether the nature of the object representation (orientation-dependent vs orientation-invariant) depends on the information-processing stages tapped by the task.

**Methodology/ Findings:**

We employed a repetition priming paradigm in which briefly presented masked objects (primes) were followed by an upright target object which had to be named as rapidly as possible. The primes were presented for variable durations (ranging from 16 to 350 ms) and in various image-plane orientations (from 0° to 180°, in 30° steps). Significant priming was obtained for prime durations above 70 ms, but not for prime durations of 16 ms and 47 ms, and did not vary as a function of prime orientation. In contrast, naming the same objects that served as primes resulted in orientation-dependent reaction time costs.

**Conclusions/Significance:**

These results suggest that initial processing of object identity is mediated by orientation-independent information and that orientation costs in performance arise when objects are consolidated in visual short-term memory in order to be reported.

## Introduction

Theories of object recognition can be broadly distinguished in terms of the claims they make about how object representations code information about shape and object orientation. Structural description models [1]–[4] assume that information about constituent parts of an object is encoded separately from information about their spatial configuration. In contrast, image-based models state that object representations consist of views in which the object parts are intrinsically related to their spatial location in the image [e.g., 5], [6]. These contrasting theoretical approaches tend to make different predictions about how an object's orientation will impact on one's ability to recognize the object and, consequently, have come to be grouped loosely into “viewpoint-dependent” and “viewpoint-invariant” classes of models. (N.B. The term ‘object orientation’ is used here to refer to the global orientation of an object; e.g, the orientation of an object's principal axis of elongation relative to a viewer-centred or environment-centred 2D reference frame, see [7], [8]). However, as Hummel [7] points out, this classification often confuses a behavioral marker (viewpoint-dependent vs viewpoint-invariant performance) with a computational issue (the nature of the object representation–image-based vs structural description) when, in fact, there is no straightforward one-to-one mapping from one to the other.

A finding often cited in support of image-based models is that recognition efficiency declines as a function of viewpoint as an object is rotated around a variety of axes from a familiar view [8], [9]. However, the observation of viewpoint costs is also compatible with structural description models [2]. Such models do not necessarily predict that recognition should be unaffected by either image plane or depth rotation, although they do predict greater generalization across small viewpoint differences that preserve the same shape features and spatial relations between them. Across larger viewpoint changes, however, most structural descriptions also predict viewpoint-dependent recognition performance.

In contrast, a range of findings suggest that object shape or identity is processed independently of the object's global orientation. Perhaps the most compelling example is neuropsychological patients who recognize objects regardless of orientation, but cannot interpret object orientation [10], [11]. Likewise, some experimental studies have found that object identity is determined before object orientation [12] while others have shown that orientation can be primed independently of shape [13]. Furthermore, viewpoint costs are not always observed and seem to depend on a variety of stimulus and task variables [14]–[16].

One important variable is the extent to which an object is attended and processed. For example, Dux and Harris [16] have shown that when multiple objects are presented one after another in the same spatial location for 100 ms each (rapid serial visual presentation [RSVP]), orientation costs are incurred for objects that are selected and encoded for report (targets), but not for the ignored distractors. It is generally accepted that all stimuli in an RSVP stream, including distractors, are identified briefly but they require additional processing if they are encoded in a more durable form [17]. Thus, Dux and Harris' [16] findings suggest that initial activation of object identity is orientation-invariant and that orientation effects arise at a later stage of encoding and consolidation in visual short-term memory (VSTM). Further support for this comes from repetition blindness studies that used objects presented in different orientations [18], [19]. This phenomenon is generally attributed to the fact that the two repetitions activate the same identity representation (*type*) but fail to be encoded as distinct visual episodes (*tokens*) [20]. Harris and Dux [18], [19] found repetition blindness for repeated objects that differed in orientation by up to 180°, consistent with the proposal that initial type activation is orientation-invariant.

These RSVP studies suggest that shape information capable of supporting orientation-invariant recognition is extracted within the first 100 ms of processing. This is somewhat surprising given that most theories of object recognition predict viewpoint costs when objects are presented only briefly. Image-based theories generally propose some kind of normalization mechanism by which the viewed image is brought into correspondence with object information stored in memory [e.g.], [9,21], which may not be able to be completed during brief presentations. More neurophysiologically inspired models suggest that evidence about an object takes longer to accumulate and reach recognition threshold when objects are presented in unfamiliar or rotated views [22]. Similarly, creating a structural description is thought to require time and visual attention; according to Hummel [23], when there is insufficient time, or when attention is directed elsewhere, recognition is mediated by holistic image-based representations in which shape features are inherently linked to their spatial locations in the image and, hence, to the orientation of the object.

In the present study, we used a repetition priming paradigm to extend our investigation of whether the initial stages of object identification, prior to consolidation in VSTM are sensitive to orientation. Several previous studies have investigated the effects of orientation on repetition priming, with mixed results. Some experiments found similar priming for identical versions of objects and their mirror reflections [e.g., 24], as well as across changes in picture-plane orientation [25], consistent with the idea that priming is orientation-invariant. In contrast, other experiments have found that the priming effects were considerably reduced by an orientation change [26]–[28]. Although these differences could have arisen for a number of reasons, including the nature of the stimuli and the tasks used, it is worth noting that practically all of these studies required explicit identification (usually in the form of naming) of both prime and target, which necessarily includes both the initial identification of a stimulus and its consolidation in VSTM. Thus, the presence or absence of orientation effects in these studies cannot be attributed specifically to either stage of processing.

For this reason, Experiment 1 employed briefly presented and masked primes, which the participant was instructed to ignore, and measured the amount of priming (i.e., facilitated naming) of subsequent targets that were either the same object as the prime, or a different object. The primes were presented for variable amounts of time (from 16 ms to 350 ms) and in different image-plane orientations (from 0° to 180°). We used stimuli rotated in the image plane because this case affords the clearest dissociation between processing of shape and orientation without the added complications of feature occlusion and shape distortions that are introduced by rotations in depth. We expected that target identification would be facilitated when it was preceded by an identical prime. The question of interest was whether this priming effect was orientation-dependent or orientation-invariant. As outlined above, most object recognition models predict orientation-dependent priming for relatively short prime durations. Similarly, the amount of priming from more rotated primes is predicted to increase as prime duration increases because there would be more time to normalize the prime, accumulate evidence about it, or derive a structural description.

The effects of orientation on later processing stages, i.e. encoding and consolidation in VSTM were evaluated in a second experiment which required speeded naming of the same objects that served as primes in Experiment 1.

## Methods

### Experiment 1: Object priming

#### Participants

102 undergraduates (mean age = 19.6 years) participated for course credit. All were native English speakers and had normal or corrected-to-normal vision. They gave written consent to participate and the procedures were approved by the Sydney University Human Research Ethics Committee.

#### Stimuli and Apparatus

Stimuli comprised 168 photographs of real objects with a well-defined canonical upright orientation from the Hemera Photo-Object collection (Hemera Technologies Inc, Canada). They were converted to greyscale and displayed against a grey background (RGB values: 190,190,190). Objects were scaled to 472 pixels in the longest dimension and subtended a visual angle of ∼9° at the viewing distance of 45cm. Pattern masks were created from collages of (unrecognizable) fragments of a number of the original pictures, cut into random shapes and superimposed in random orientations.

Stimuli were displayed on a 19″ CRT monitor with vertical refresh rate of 85 Hz, or 120Hz (16 ms prime exposure group). The experiment was constructed and run using DMDX [29].

#### Design

The priming task used a (2×7)×6 design, with Prime Identity (Same vs. Different prime) and Prime Orientation (0°, 30°, 60°, 90°, 120°, 150°, 180°) as within-subject factors and Prime Duration (16 ms, 47 ms, 70 ms, 95 ms, 141 ms, 350 ms) as a between-subjects factor. There were 15 subjects in each prime duration group, except for 47 ms and 70 ms, where we tested 21 subjects.

The objects were divided randomly into three groups (A, B, C) which were used to create three versions of the experiment. Version 1 contained A-A object pairings in the Same prime trials and B-C pairings in the Different prime trials; Version 2 contained B-B pairings in the Same prime trials and A-C pairings in the Different prime trials, while Version 3 contained C-C pairings in the Same prime trials and A-B pairings in the Different prime trials. This ensured that each prime only appeared once in the experiment, given that presenting rotated objects repeatedly is known to diminish viewpoint effects [8]. For each group of primes, 8 objects were randomly allocated to each of the 7 orientations; with the exception of the 0° and 180° conditions, at each orientation 4 objects were rotated clockwise and 4 counter-clockwise. Each participant completed one version of the experiment, comprising 112 trials (56 same prime trials and 56 different prime trials) in a randomly intermixed order.

#### Procedure

Participants sat approximately 45cm from the monitor and responded verbally using a microphone connected to a voice key which recorded voice onset. The experimenter verified response accuracy and noted spoilt trials for later removal. Before the experiment, participants completed a familiarization phase in which they saw all objects in their normal upright orientations and named them at their own pace. Feedback was given for all incorrect or ambiguous names (e.g., “shoe” was not allowed for other footwear, such as boot).

**Figure 1:**
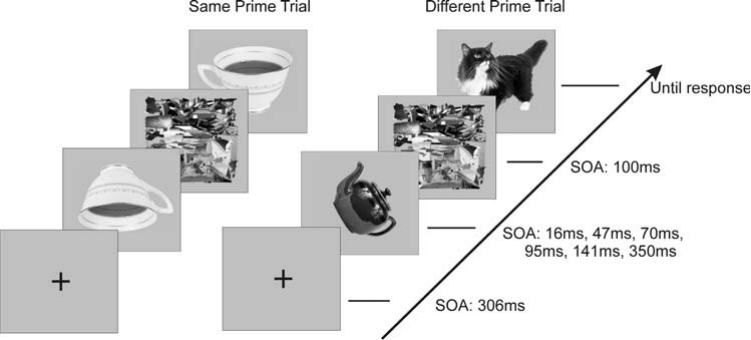
Examples of experimental trials. On half the trials, the prime was the same object as the target and on the other half it was a different object. The prime could be in one of 7 orientations ranging from 0° to 180° in 30° steps, while the target was always upright. The duration of the prime was varied by varying the prime-mask SOA.

Each trial began with a fixation cross for 306 ms, followed by the prime with a variable duration, followed by a pattern mask for 100 ms, and then by the target picture which remained on the screen until response or for a maximum of 2s (see Fig. 1). The prime could be in one of 7 orientations between 0° and 180°, whereas the target was always upright. Participants were instructed to attend to the target object and to name it as quickly and as accurately as possible. They were told that another object will flash briefly before this, together with a mask, but that they need not pay any attention to it. The experimental trials were preceded by 10 practice trials using different objects.

### Experiment 2: Object Naming

#### Participants

Fifteen undergraduates, aged 18–42 years (mean = 21) participated for course credit. They were all native English speakers and had not participated in the previous experiment. They gave written consent to participate and the procedures were approved by the Sydney University Human Research Ethics Committee.

#### Procedure

In terms of experimental design, this experiment was an exact replica of Experiment 1, with the mask and the upright second object removed from each trial. Thus, subjects named a total of 16 objects in each of the 7 orientations, with half the items in each orientation rotated clockwise and half counter-clockwise. There was no familiarization phase prior to the experiment, and so objects were seen and named for the very first time during the experimental trials. For this reason, the pictures were displayed for a maximum of 3s. All other procedural details were as in Experiment 1.

## Results

### Experiment 1: Object priming

Spoilt trials (e.g., premature triggering of the microphone) and trials in which no response was recorded within 2s were eliminated from further analysis. Accuracy was high (overall mean 95%), and did not differ according to prime identity or orientation (Fs<1.13, ps>.29, η^2^<.05). There was an effect of prime duration, F(5,96) = 18.80, p<.001, η^2^ = .50, with lower accuracy for the 16 ms prime duration compared to the other durations (see Table 1).

**Table 1:** Summary of overall priming effects and accuracy levels in Experiment 1, averaged across orientations.

*Prime Duration*	*Mean size of priming effect (SD)*	*Accuracy level (SD)*
16 ms	−9.39 (37.50)	88% (12.60)
47 ms	9.50 (37.98)	97% (7.20)
70 ms	19.16 (35.10)^*^	96% (7.30)
95 ms	35.81 (51.86)^*^	99% (4.20)
141 ms	66.31 (56.05)^#^	97% (6.21)
350 ms	113.25 (56.56)^#^	94% (9.07)

Note: Size of priming effect (ms) = RT on Different prime trials-RT on Same prime trials. ^*^p <.05, ^#^p<.001

Mean reaction times (RT) for correct responses are displayed in Table 2 and were subjected to a mixed-design ANOVA with Prime Duration as a between-subject factor and Prime Identity and Prime Orientation as repeated measures. First of all, the overall level of performance did not differ amongst Prime Duration groups, F(5,96) = 1.18, p = .33, η^2^ = .06. There was a significant main effect of Prime Identity, F(1,96) = 73.19, p<.001, η^2^ = .43, qualified however by a significant Prime Identity×Prime Duration interaction, F(5,96) = 14.90, p<.001, η^2^ = .44, which indicates that the magnitude of the priming effect increased with increasing prime duration (see Table 1). There was also an overall effect of Prime Orientation, F(6,30) = 2.57, p = .02, η^2^ = .026, but no significant Prime Identity×Orientation interaction, F(6,30) = 1.63, p = .14, η^2^ = .017, nor a significant 3-way interaction, F<.51. This suggests that the priming effect was not modulated by object orientation at any prime duration.

**Table 2:** Mean target naming times (and SD) across prime duration and orientation conditions in Experiment 1.

Prime Duration	Orientation						
	0°	30°	60°	90°	120°	150°	180°
*16 ms*							
Same prime	905 (136)	917 (130)	887 (118)	910 (103)	925 (122)	896 (114)	900 (128)
Different prime	909 (149)	862 (106)	890 (138)	921 (131)	899 (105)	899 (118)	895 (115)
*47 ms*							
Same prime	866 (99)	862 (126)	855 (134)	876 (129)	847 (128)	888 (119)	837 (118)
Different prime	857 (117)	881 (142)	881 (136)	870 (125)	860 (136)	867 (137)	881 (128)
*70 ms*							
Same prime	886 (126)	886 (130)	869 (107)	897 (130)	886 (126)	896 (117)	880 (81)
Different prime	880 (117)	899 (107)	918 (140)	924 (117)	903 (90)	900 (130)	911 (129)
*95 ms*							
Same prime	889 (123)	933 (116)	910 (126)	933 (118)	911 (126)	954 (155)	884 (85)
Different prime	942 (105)	954 (146)	951 (125)	975 (158)	970 (108)	925 (125)	948 (160)
*141 ms*							
Same prime	820 (168)	854 (165)	797 (141)	869 (174)	823 (151)	850 (190)	844 (171)
Different prime	880 (132)	920 (172)	899 (121)	900 (124)	927 (139)	908 (141)	885 (116)
*350 ms*							
Same prime	805 (73)	815 (114)	772 (99)	845 (118)	826 (123)	844 (153)	798 (88)
Different prime	911 (74)	912 (92)	930 (127)	930 (84)	951 (138)	938 (81)	928 (75)

**Figure 2:**
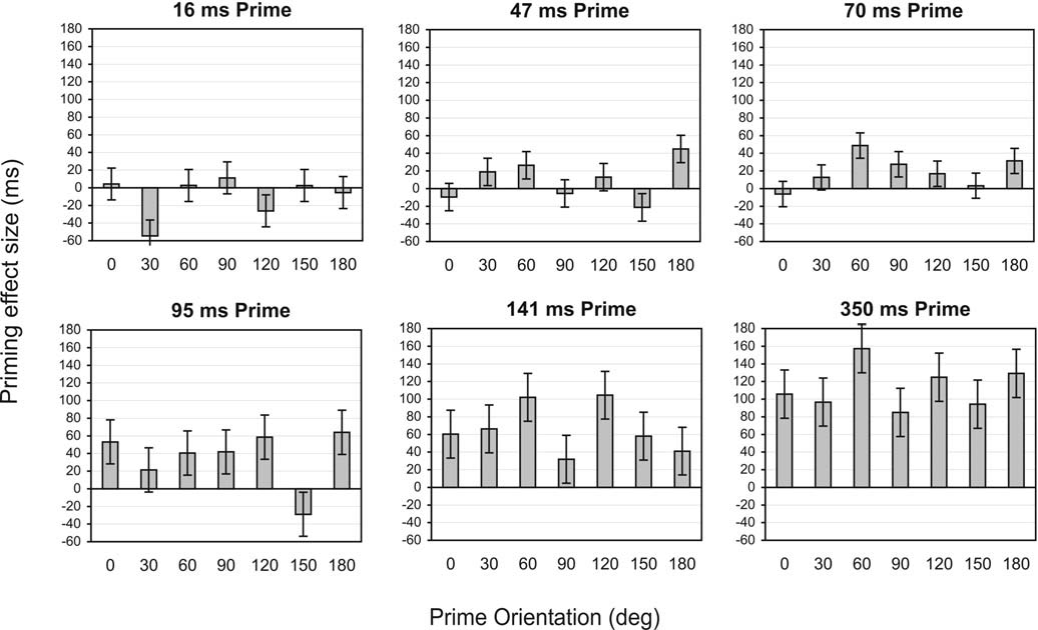
Mean priming effect in Experiment 1, plotted as a function of prime orientation for each prime duration separately. The size of the priming effect is the difference between RTs for Different prime trials and RTs for Same prime trials. Error bars represent within-subject s.e.m for the priming effect.

Separate 2 (Prime Identity)×7 (Orientation) repeated-measures ANOVAs were performed on the data for each prime duration condition. These revealed significant effects of Prime Identity for prime durations of 70 ms and greater (Fs>6.27, ps<.05, η^2^>.24), but not for prime durations of 16 ms or 47 ms (Fs<1.32, ps>.27, η^2^<.06)–see Table 1 for priming magnitude in each condition. There were no significant main effects of Prime Orientation or interactions between Prime Identity and Orientation for any prime duration (all Fs<1.30, ps>.27, η^2^<.084). These results are illustrated in Figure 2.

### Experiment 2: Object Naming

**Figure 3:**
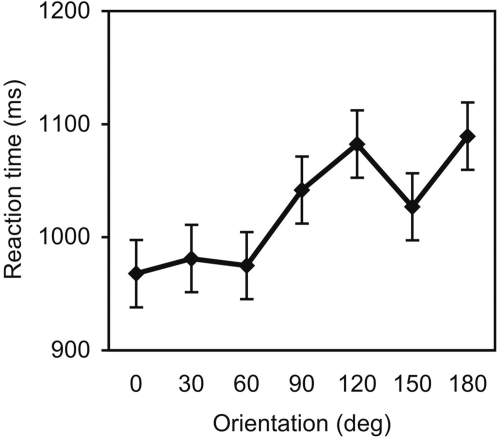
Mean reaction times for naming rotated objects in Experiment 2, plotted as a function of object orientation. Error bars represent within-subject s.e.m for the orientation effect.

Naming accuracy was reasonably high despite the lack of practice and feedback (88% correct overall, ranging across orientations from 84–92%). There was no effect of orientation on accuracy (F<1). Mean RT for correct responses are shown in Figure 3 and clearly indicate an orientation-dependent pattern of RT, which increased systematically as a function of orientation. Analyses confirmed a significant linear trend in the data, F(1,14) = 9.23, p<.01, η^2^ = .40, with no higher-order components. The slope of the naming function was 0.67 ms/deg. Thus, on average, there was a cost of 121 ms when naming objects rotated by 180°, compared to upright objects.

## Discussion

This study investigated the effects of exposure duration and orientation of an ignored prime on subsequent naming of an upright object, in order to test whether the early stages of object processing (i.e., prior to encoding and consolidation in VSTM) are sensitive to the image-plane orientation of the object. This was contrasted with the effects of orientation for objects that undergo consolidation for report. There are three findings of interest. First, the amount of repetition priming increased as prime duration increased. In this study, a prime duration of 70 ms yielded a reliable priming effect across subjects, indicating that this is enough time to extract information that can lead to successful object identification.

Second, and most importantly, the amount of priming was independent of prime orientation, regardless of prime duration. These results are consistent with our previous RSVP studies of object recognition which showed that initial type (i.e., identity representation) activation is orientation-invariant [16], [18], [19]. A similar finding was also reported by Murray [30], who found orientation-invariant negative priming from actively ignored objects that were semantically related to the target. Our present findings, however, contradict those of a related repetition priming study conducted by Arguin and Leek [31], in which a brief prime presented in different image-plane orientations was followed, after a variable blank period, by an upright target that had to be named. Arguin and Leek found that if the stimulus onset asynchrony (SOA) separating prime and target was 1s long, priming was orientation-invariant. For short SOAs (100 and 200 ms), however, they found preferential priming from upright primes compared to rotated primes, and for an SOA of 500 ms the size of priming increased linearly across the three prime orientations (0°, 90°, 180°). Arguin and Leek interpreted these results as evidence for a normalization process that was still partly incomplete at 500 ms SOA. A problem with this interpretation is that the RT costs associated with identification of rotated objects are typically in the range of 100–250 ms for the most time-consuming orientations [e.g., 8], [32, and also the present study] and thus any putative normalization of rotated primes should have been well and truly completed within the 500 ms SOA, and even within the shorter SOA of 200 ms. We suspect that the discrepancy between their findings and the present ones is due to Arguin and Leek not using a mask between the prime and the target, which may have created some undesirable differences between their conditions. Specifically, at short SOAs an upright prime followed by the identical upright target would have most likely appeared as one continuous stimulus due to the iconic memory trace of the prime [33]. In contrast, a rotated prime followed by an upright target would have engendered a very obvious transition, making it clear that these were two different stimuli and likely slowing down response to the target. This may account for the orientation-dependent priming seen in that study.

Third, the orientation-invariant priming obtained from an unattended prime was in sharp contrast to the orientation-dependent naming performance found in Experiment 2, where participants attended to and named the same objects that served as primes in the first experiment. In this context, it is interesting to note that a prime duration of 350 ms, which one would expect to be long enough to enable conscious identification, did not give rise to orientation-dependent priming. This suggests that the orientation-dependent effects seen in Experiment 2 are specifically associated with active selection and consolidation in a more lasting form, rather than with the activation of identity representations in memory [see also 16].

In broader theoretical terms, the present results pose a challenge for image-based models of object recognition. As outlined earlier, although these models differ in the mechanisms invoked to explain orientation costs (e.g., spatial normalization or alignment, evidence accumulation, mental rotation, etc), collectively they claim that shape features are inherently bound to their spatial location in the image and, hence, to object orientation. As such, these models would predict priming only for identically oriented (i.e., upright) prime-target pairs for short prime durations, with correspondingly smaller amounts of priming for more rotated primes, as compensation for object misorientation (whatever form that takes) could not be completed in the time available. Contrary to these predictions, we did not find preferential priming for upright prime-target pairs compared to other orientations even for very short prime durations (see Fig. 2).

Interestingly, these findings are also inconsistent with some current structural description models. These models predict less or no orientation costs for relatively small rotations which preserve parts and their spatial relations (and, therefore, the same structural description), but they do predict costs for larger rotations which perturb the spatial relations between parts [2], [23]. Thus, these theories would also have difficulty accounting for the complete orientation-invariant priming demonstrated in the present study. The findings are also inconsistent with a recent “hybrid” model proposed by Hummel and his colleagues [23], [34], [35]. This model states that in the absence of attention, recognition is mediated by holistic (and orientation-dependent) representations, whereas a structural description that specifies object parts independently of spatial information can be derived under conditions of full attention. Our finding of orientation-invariant priming from an ignored prime clearly challenges this account.

One plausible explanation for our results is that priming in this paradigm is mediated by local shape features or parts, independently of their spatial relation (i.e., prior to deriving a structural description). Alternatively, priming could occur at a relatively coarse (and orientation-invariant) level of processing, sufficient for object categorization (e.g., as an animal), but not for specific identification (e.g., as a cat). The present study was not specifically designed to distinguish between these two alternatives and this must await further investigation. However, the present findings support the notion that the initial stages of object recognition, prior to consolidation in VSTM, are insensitive to object orientation and that orientation effects arise at a later stage of processing when objects are attended and consolidated for report.
